# Decoding immune low-response states in sepsis: single-cell and 3D spatial transcriptomic insights into immunoparalysis

**DOI:** 10.3389/fimmu.2025.1696914

**Published:** 2025-11-05

**Authors:** Yulian Yang, Yi Zhang, Jingjing Wu, Yi Liu, Xianying Lei

**Affiliations:** ^1^ Department of Critical Care Medicine, The Affiliated Hospital, Southwest Medical University, Luzhou, Sichuan, China; ^2^ Department of Critical Care Medicine, The Second People’s Hospital of Deyang, Deyang, Sichuan, China

**Keywords:** sepsis, immunoparalysis, immune low-response states, monocyte HLA-DR, endotoxin tolerance, single-cell RNA sequencing

## Abstract

Sepsis remains a leading cause of critical illness worldwide. Despite advances in supportive care, durable benefit from immune-directed therapies is limited, reflecting heterogeneity with immune low-response states (‘immunoparalysis’) across innate and adaptive compartments. In this review we summarize advances from single-cell RNA and ATAC profiling, immune-repertoire assays and 3D spatial transcriptomics that resolve monocyte, dendritic-cell (cDC1, cDC2 and pDC), lymphocyte and NK-cell programs, and appraise translational opportunities spanning endotype-guided risk stratification, pharmacodynamic monitoring and spatial biomarkers. We also discuss enduring challenges—including assay standardization, harmonized thresholds for monocyte HLA-DR and whole-blood stimulation, and limited availability of clinically compatible spatial platforms—that temper implementation. By integrating bedside function (HLA-DR trajectories, LPS-induced cytokine capacity) with single-cell endotypes (MS1/HLA-DR^low S100A^high monocytes, dendritic-cell attrition, checkpoint-biased T cells) and host–pathogen topology from FFPE-ready spatial assays, emerging strategies aim to restore antigen presentation, reconstitute priming, disrupt inhibitory myeloid–lymphoid circuits and prevent secondary infection. Our synthesis provides an appraisal of the evolving landscape of immunoparalysis-informed precision medicine in sepsis and outlines pragmatic standards for composite biomarkers, patient selection and on-therapy decision rules. We hope these insights will assist investigators and clinicians as they endeavor to convert descriptive immune low-response states into tractable, reversible clinical entities.

## 1 Introduction

Sepsis is a leading cause of critical illness and death worldwide and is characterized by organ dysfunction arising from a dysregulated host response to infection (1–4). Contemporary immunology has reframed this response as dynamic and heterogeneous, with phases of exuberant inflammation often overlapped by immune low-response states collectively termed immunoparalysis (5–7). These states are marked by impaired antigen presentation, altered cytokine production, and defects in innate–adaptive crosstalk that increase susceptibility to secondary infection and adverse outcomes, underscoring the need for precise endotyping and immune-directed interventions (8–11).

Clinically, immunoparalysis is captured by functional and phenotypic readouts that indicate reduced host defense capacity. Two complementary assays are most established: ex vivo lipopolysaccharide–stimulated cytokine production (typically diminished tumor necrosis factor-α release) and decreased expression of HLA-DR on circulating monocytes, the latter indexing an antigen-presentation deficit (12–15). Both correlate with infection risk and mortality and have been proposed for risk stratification and for selecting patients into immuno-adjuvant trials (16–18). Mechanistically, these abnormalities align with endotoxin tolerance programs in myeloid cells and checkpoint-mediated exhaustion in lymphocytes.

High-dimensional profiling now offers a path to resolve this heterogeneity. Single-cell RNA sequencing in sepsis blood defined discrete immune states—including an expanded CD14^+^ monocyte state with suppressed HLA-DR and altered inflammatory signaling—that robustly distinguish patients and provide mechanistic anchors for biomarker development (19–21). Subsequent multicohort analyses reinforced that composite single-cell signatures capturing lymphopenia, dendritic-cell loss, and myeloid HLA-DR downregulation track disease trajectories and may improve diagnostics and prognostication across age groups (22–24). These findings support an endotype-based view of sepsis in which therapeutic responsiveness depends on the prevailing immune program rather than on a uniform “hyperinflammation” construct.

At the tissue level, dissociative assays incompletely represent microanatomical context, which is critical for understanding host–pathogen interfaces, vascular compromise, and compartmentalized immunoregulation in sepsis. Spatially resolved transcriptomics has therefore emerged as a complementary approach, recognized for enabling quantitative maps of gene expression within intact architecture; recent advances extend these maps into three dimensions, providing volumetric views of cellular neighborhoods and gradients relevant to barrier defense and organ injury (25–28). The integration of spatial readouts with single-cell state dictionaries creates opportunities to localize immunoparalysis niches, quantify cell–cell communication *in situ*, and nominate spatial biomarkers suitable for translation.

This review synthesizes evidence on immune low-response states in sepsis with a focus on how single-cell modalities (scRNA-seq, scATAC-seq, CITE-seq, TCR/BCR profiling) and three-dimensional spatial transcriptomics delineate innate–adaptive programs, tissue topology, and host–pathogen contact zones. The aim is to standardize concepts and measurement frameworks for immunoparalysis, highlight robust cellular and spatial biomarkers for risk stratification, and outline principles for patient selection and pharmacodynamic monitoring in trials that seek to reverse immune low-response states.

## 2 Defining immunoparalysis in sepsis: concepts, metrics, and clinical context

**Figure 1:**
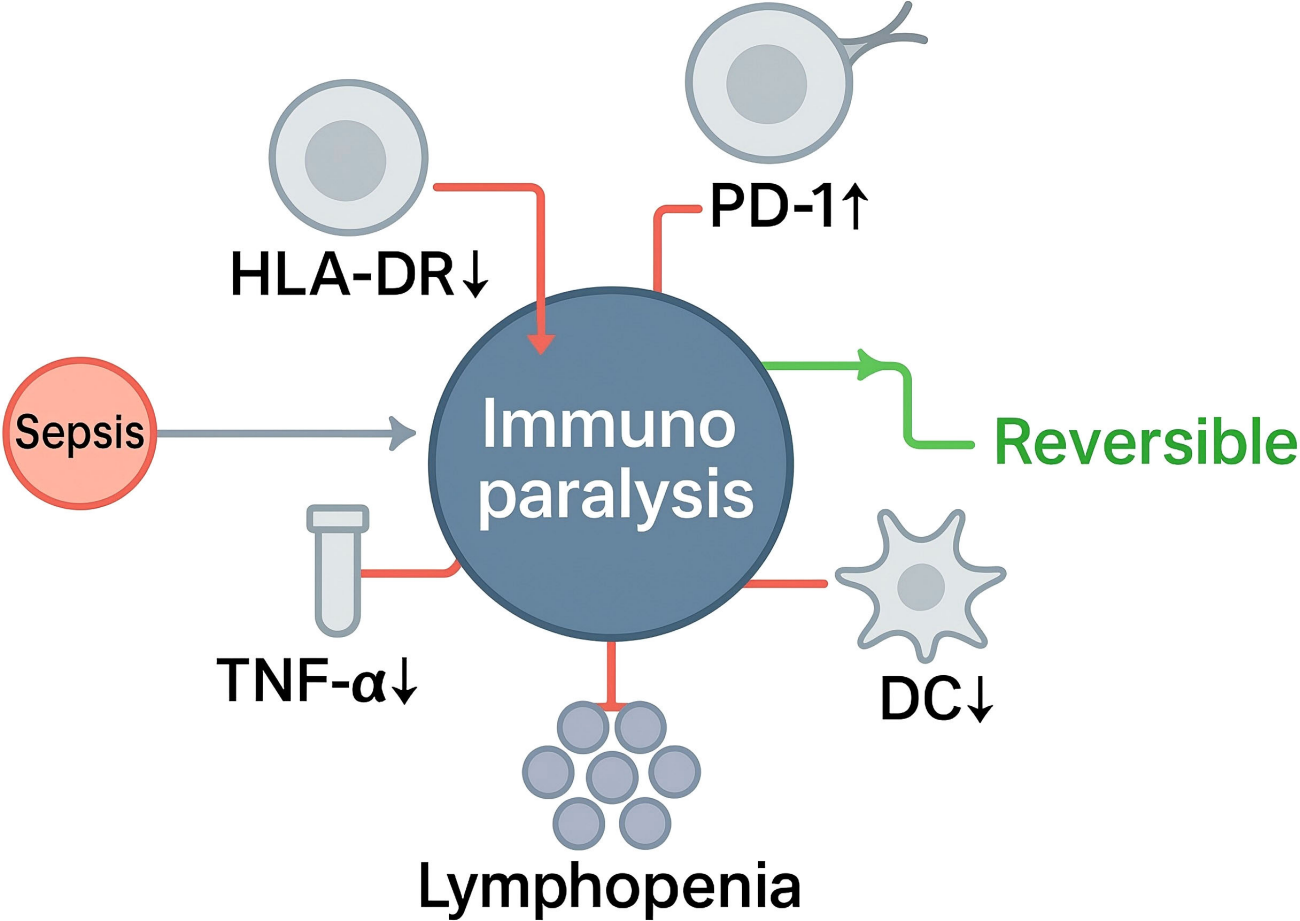
Sepsis-induced immunoparalysis: key reversible hallmarks.

As shown in **Figure 1**, immunoparalysis denotes a clinically significant, often reversible, low-response immune state that arises during sepsis and is characterized by concurrent defects across innate and adaptive compartments, including impaired antigen presentation, blunted stimulus-induced cytokine production, apoptosis-associated lymphocyte depletion, and checkpoint-mediated T-cell dysfunction (29–31). These abnormalities reflect an adaptive reprogramming frequently described as endotoxin tolerance in myeloid cells and exhaustion or anergy in lymphocytes, and they contribute to heightened susceptibility to secondary infection and adverse outcomes. Conceptually, this state is supported by mechanistic and clinical observations of reduced HLA-DR expression on antigen-presenting cells, diminished ex vivo cytokine release after lipopolysaccharide challenge, quantitative and qualitative lymphocyte defects, and subset-specific dendritic-cell abnormalities—depletion of cDC1, functional impairment of cDC2, and contraction of pDCs (markers as listed), with the same transcriptional programs and signaling changes as previously described.

Operationalization in practice relies on complementary functional and phenotypic readouts. Monocyte HLA-DR measured by standardized flow cytometry is the most widely adopted marker of innate deactivation; persistently low expression identifies patients at increased risk of nosocomial infection and death and has been proposed as an indicator of “immune organ failure” suitable for risk stratification and for selecting candidates for immunostimulatory interventions. Longitudinal trajectories of HLA-DR during septic shock refine this risk assessment and capture recovery versus persistent suppression (32–35). Functional assays—CD107a degranulation (K562 or PMA/ionomycin) and IFN-γ release after IL-12/IL-18—map to risk (lower responses predict secondary infection) and serve as on-therapy targets with LPS-TNF and HLA-DR (36–38). Adaptive immune components provide parallel indicators: global lymphopenia and features of T-cell dysfunction, including increased PD-1/PD-L1, LAG-3 and TIM-3 signaling, align with impaired host defense and are being explored as therapeutic targets in biomarker-enriched trials (39–41). Dendritic-cell loss and dysfunction further reduce antigen presentation capacity and have been linked to secondary infection risk in septic shock, validating their inclusion within a composite definition (42, 43). In malignancy, CKD, diabetes, or prior immunosuppression, interpret ‘low-response’ using within-patient deltas and percentile thresholds, and require concordance across ≥2 modalities (e.g., HLA-DR^low^ plus LPS-TNF^low^).

A practical definition of immunoparalysis in sepsis is a time-varying syndrome of impaired innate and adaptive immunity evidenced by persistently low monocyte HLA-DR, reduced stimulus-induced cytokine production capacity, lymphocyte depletion and/or exhaustion signatures, and dendritic-cell deficiency, each associated with elevated risk of secondary infection and mortality. This framework supports standardized endotyping and provides an entry point for biomarker-guided, immune-restorative strategies in critical illness. Secondary infection’ denotes a new, adjudicated infection ≥48 h after index presentation (or after initial control), assessed in day-14 and day-28 windows.

## 3 Single-cell dissection of immune low-response states: innate–adaptive programs and regulatory circuits

Single-cell modalities resolve the composite nature of immune low-response states by jointly defining cell identities, activation gradients, and regulatory dependencies at single-cell resolution. Across independent cohorts, scRNA-seq consistently identifies an expanded myeloid program characterized by HLA-DR suppression and increased S100A8/A9, RETN, VCAN, and IL1R2 expression—often referred to as the “MS1/HLA-DR^low^ S100A^high^” monocyte state—which is mechanistically linked to impaired antigen presentation and broad inhibitory crosstalk with lymphocytes and dendritic cells (e.g., predicted LGALS9–HAVCR2 and class I HLA–LILRB interactions) (44–46). These features recapitulate clinically recognized innate deactivation and provide a cell-state framework for immunoparalysis endotyping, as detailed in **Table 1**.

**Table 1:** Key single-cell–defined immune low-response programs in sepsis and how to recognize them.

Program/Cell State	Canonical transcript or protein features (illustrative)	Single-cell readouts that establish the state	Representative inhibitory signals or network features	Functional interpretation for immunoparalysis	Analytic notes (quality and integration)
HLA-DR^low^ S100A^high^ monocytes (“MS1”-like)	↓HLA-DRA/DRB1, ↓CD74; ↑S100A8/S100A9, RETN, VCAN, IL1R2, LILRB1/2	scRNA-seq clusters with antigen-presentation deficit; CITE-seq confirms low HLA-DR surface protein	Predicted LGALS9–HAVCR2, HLA class I–LILRB, TGFB1–TGFBR pathways; myeloid suppressive regulons	Innate deactivation; antigen presentation failure; broad suppression of lymphocyte function	Guard against neutrophil contamination; harmonize ambient RNA removal; validate with flow cytometry
Antigen-presenting monocytes/DC with reduced capacity	↓HLA-class II genes; ↓CCR7; ↓CD86	Depletion of cDC1/cDC2 clusters; reduced co-stimulatory gene modules	Weak ligand delivery to T cells; impaired IL-12/IFN axes	Impaired priming and T-cell activation	Include whole-blood or DC-enriched sampling; standardize batch correction across centers
Exhausted/dysfunctional CD8^+^ and CD4^+^ T cells	↑PDCD1, LAG3, TIGIT, HAVCR2; ↓IL7R; reduced cytotoxic module in subsets	scRNA-seq state gradients; TCR clonality skew; diminished effector gene scores	Inhibitory checkpoint circuits; reduced antigen-receipt signatures	Blunted effector responses; susceptibility to secondary infection	Pair scRNA-seq with TCR-seq; control for lymphopenia when comparing proportions
Atypical memory/age-associated B cells	↑ITGAX (CD11c), FCRL5, TBX21; altered SHM/CSR patterns	BCR clonotype contraction; scRNA-seq B-cell state shift	Attenuated antigen presentation to T cells	Suboptimal antibody responses and help to T cells	Joint BCR-seq profiling advisable; remove doublets (B/T conjugates)
NK cell hyporesponsiveness	↑KLRC1 (NKG2A); ↓GNLY, PRF1, NKG7 in subsets	Reduced cytotoxicity modules; altered cytokine gene scores	Inhibitory receptor dominance; reduced activating ligand inputs	Weakened early pathogen control	Include whole-blood captures; verify with degranulation markers if available
Myeloid progenitor bias/trained-tolerance imprint	Accessibility shifts at myeloid enhancers; ↑C/EBP/STAT motif activity	scATAC-seq peaks linked to tolerized genes; integrated GRN modules	Stabilized tolerized gene programs; diminished inflammatory transcription	Persistence of hyporesponsive myelopoiesis	Integrate scATAC-seq with scRNA-seq; use batch-aware peak calling
Cross-talk bottlenecks (myeloid→lymphoid)	Diminished costimulatory ligands; dominance of inhibitory ligands	Ligand–receptor networks showing low CD28/ICOS signaling and high inhibitory pairs	Net negative signaling into T/NK compartments	Propagation of low-response state across compartments	Apply curated LR databases; adjust for cell-type composition effects

↓, decreased/downregulated expression/abundance; ↑, increased/upregulated expression/abundance.

Adaptive compartments show concurrent low-response programs detectable by scRNA-seq and immune-repertoire profiling. Across cohorts, cDC1 depletion occurs earliest and most profoundly with down-shifted CLEC9A/XCR1 and BATF3/IRF8 regulons; cDC2 persist but exhibit dysfunction with impaired CD86/CCR7 and IL12B modules; and pDCs contract with attenuated IRF7 programs and type I interferon release—together explaining suboptimal priming and Th1 skewing. Cytokine production capacity and antigen-presentation cues decline in parallel with transcriptional features of T-cell dysfunction, including enrichment of inhibitory checkpoint transcripts and contraction of naïve/central memory pools, while dendritic-cell fractions decrease (47–49). These single-cell readouts align with clinical low-response phenotypes and reveal trajectories from activation to dysfunction that are not captured by bulk assays.

Regulatory-circuit reconstruction strengthens mechanistic inference. Chromatin accessibility profiling (scATAC-seq) and integrated analyses of endotoxin-tolerance–like states identify promoter–enhancer reconfiguration and transcription-factor programs that stabilize monocyte hyporesponsiveness, while multimodal pipelines infer active regulons and state transitions that track movement toward low-response phenotypes (50–52). Dynamic modeling (RNA velocity) and ligand–receptor inference systematically connect myeloid inhibitory signaling to T- and NK-cell dysfunction—ideally using composition-preserving permutations or cell-count offsets to control compositional confounding, zero-inflation–aware models to mitigate gene dropout, and FDR correction for multi-test burden—offering testable hypotheses for pharmacodynamic reversal.

Tissue-level sampling extends these insights beyond blood by localizing low-response programs within injured organs and host–pathogen contact zones (53–55). Time-resolved single-cell maps in septic organs reveal compartmentalized myeloid reprogramming, dendritic-cell attrition, and disrupted lymphocyte niches, supporting the view that immunoparalysis is a distributed, topology-dependent state that can now be quantified and monitored with single-cell tools.

The above cell-state dictionary supports a practical approach to endotyping: prioritize detection of the MS1/HLA-DR^low^ S100A^high^ monocyte expansion and dendritic-cell loss, quantify checkpoint-biased T-cell states with concurrent repertoire features, infer inhibitory myeloid–lymphoid communication, and, where feasible, corroborate stabilizing chromatin programs. These readouts provide mechanistic anchors for biomarker-guided patient selection and pharmacodynamic monitoring in trials that seek to reverse immune low-response states.

## 4 3D spatial transcriptomics of sepsis tissues: ecosystem topology and host–pathogen interfaces

Three-dimensional spatial transcriptomics enables quantitative mapping of immune activity within intact organ architecture, providing volumetric context for gradients, barriers, and interfacial zones that shape antimicrobial defense during sepsis. Organism-wide spatial profiling in experimental sepsis already demonstrates tissue-specific transcriptional programs across multiple organs, establishing the feasibility and biological value of spatially resolved readouts for systemic infection (56–58). Extending these approaches into true 3D volumes allows precise localization of low-response immune niches at epithelial, endothelial, and perivascular interfaces where host–pathogen contact and microcirculatory compromise converge.

Volumetric reconstruction is now technically routine through serial-section acquisition coupled to dedicated alignment frameworks. Open-ST registers consecutive sections into a coherent 3D representation while preserving whole-transcriptome coverage, enabling reconstruction of cellular neighborhoods and long-range axes within diseased tissue. Computational toolkits such as Spateo further support 3D gradient modeling and inference of intercellular interactions across entire organs (59–61). Slice-to-slice and cross-modality alignment at scale can be performed with STalign, which maps sections to a three-dimensional common coordinate framework using diffeomorphic metric mapping, and with SLAT, a graph-based algorithm that robustly aligns heterogeneous spatial slices across technologies (62–64). Together, these methods permit organ-level assemblies in which immune programs can be quantified as continuous fields rather than isolated 2D snapshots.

Within such volumes, ecosystem topology can be defined by combining cell-type deconvolution with spatial statistics that test whether ligand–receptor signaling is non-randomly organized in space (58, 65, 66). Approaches like SpatialDM use bivariate spatial autocorrelation to score interaction hotspots, allowing *in situ* quantification of immunoregulatory circuits (for example, inhibitory myeloid–lymphoid signaling) and their relationship to distances from vasculature, airspaces, or tubular lumina (28, 50, 67). This framework operationalizes “immunoparalysis niches” as measurable 3D entities that co-vary with tissue gradients relevant to sepsis pathophysiology.

Resolving host–pathogen interfaces requires simultaneous detection of microbial and host transcripts. Two sequencing-based strategies now enable this. Spatial host–microbiome sequencing (SHM-seq) co-captures polyadenylated host mRNA and 16S rRNA from bacteria on the same array, accurately mapping bacterial biogeography against host programs (68–70). Spatial metatranscriptomics (SmT) broadens this to include fungal taxa by jointly sequencing 16S and 18S/ITS, while explicitly addressing contamination risk by leveraging spatial patterns to separate true tissue-embedded microbial signals from environmental noise (71–73). For clinical material, dual spatial transcriptomics in FFPE sections achieves unbiased co-detection of human and viral RNAs, offering a practical route to archived sepsis tissues and high-containment samples. Collectively, these methods transform microbe–host colocalization from proxy histology into transcriptome-wide, spatially explicit measurements.

Platform choice influences detection at the host–pathogen boundary. Oligo-dT capture alone is suboptimal for many microbes and can be hindered by FFPE-induced 3′ tail modification, motivating total-RNA or targeted capture schemes in infected tissue (74, 75). Recent array chemistries using random primers extend spatial capture to total RNA in FFPE while retaining high spatial resolution, improving the likelihood of detecting microbial transcripts alongside host responses in clinically processed sepsis samples (76–78). High-field-of-view platforms such as Stereo-seq additionally support wide-area mapping at near-single-cell granularity, facilitating reconstruction of large infected regions and their immunoregulatory gradients.

Integration with single-cell atlases strengthens mechanistic interpretation. Projecting single-cell–defined immune states into 3D spaces using tools such as scHolography reconstructs cellular neighborhoods and refines estimates of cell–cell communication in volumes, complementing spot-level deconvolution and enabling hypothesis testing on how myeloid deactivation, dendritic-cell attrition, or checkpoint-biased T cells are spatially organized relative to pathogen density and tissue microanatomy (79–81). These analyses can be standardized across cohorts by aligning multi-slice datasets into common coordinate systems with STalign or SLAT, supporting comparative studies of therapeutic modulation of low-response states.

A practical sepsis workflow therefore acquires serial sections from infected organs, reconstructs 3D transcriptomic volumes with Open-ST or Spateo, overlays host–pathogen colocalization via SHM-seq or SmT (FFPE-compatible where necessary), and quantifies spatially significant signaling using methods such as SpatialDM. The resulting volumetric maps delineate immunoregulatory niches at barrier and vascular interfaces that are likely to determine secondary-infection risk and therapeutic responsiveness, providing a foundation for spatial biomarkers and pharmacodynamic readouts in trials seeking to reverse immune low-response states.

## 5 Clinical translation and outlook: biomarkers, risk stratification, and therapeutic reversal strategies

Clinical translation of immune low-response states in sepsis should converge on composite, serially measurable biomarkers that couple circulating function, single-cell endotypes, and spatial context to decision-making. A pragmatic framework links three tiers. First, bedside-accessible functional and phenotypic readouts quantify host-defense capacity and its recovery dynamics; thresholds and slopes over the first week should inform risks of secondary infection, prolonged organ support, and death, and gate entry into immune-restorative interventions. Second, single-cell state dictionaries—capturing expansion of HLA-DR^low^ S100A^high^ monocytes, dendritic-cell attrition, and checkpoint-biased T cells—can be operationalized as reduced-gene RNA or protein panels with predefined quality controls; these signatures provide mechanistic enrichment for trials and pharmacodynamic anchors to confirm on-target immune reversal. Third, spatially aware metrics from routine histology–compatible platforms (including FFPE-adapted spatial assays) should localize immunoparalysis niches at epithelial, endothelial, and perivascular interfaces and quantify adjacency rules (e.g., macrophage–endothelium interface length, lymphoid aggregate burden) that modify risk beyond cell fractions (24, 82–84). Integration across tiers yields an “immunoparalysis index” with calibrated cut points, validated in prospective cohorts, and accompanied by minimal-surrogate surrogates (e.g., monocyte HLA-DR plus reduced-gene myeloid signature) for resource-limited settings.

Therapeutic reversal strategies should be biomarker-guided and time-sensitive: candidates include antigen-presentation upregulation and dendritic-cell reconstitution, targeted disruption of inhibitory ligand–receptor circuits, and context-aware checkpoint modulation; for PD-1/PD-L1, LAG-3 or TIM-3 trials, enroll checkpoint-high patients (e.g., PD-1^hi^ CD8^+^ T cells and/or PD-L1^hi^ monocytes in the top cohort quartile together with HLA-DR^low), and predefine pharmacodynamic reversal as increased TCR- or cytokine-stimulated IFN-γ/TNF with higher CD107a degranulation/cytotoxic scores and a fall in checkpoint MFI or gene scores toward reference ranges (85–88). Trial designs should embed adaptive enrichment, early futility based on target engagement, and safety monitoring for hyperinflammatory breakthroughs; we recommend primary endpoints of infection-free and organ-support–free days to day-28 (composite ventilator-, vasopressor- and renal-replacement–free days), with secondary endpoints including 28/90-day mortality, new secondary infection by day-14, ΔSOFA to day-7, ICU-free days to day-28, and patient-reported outcomes. Immunostimulatory therapies should be withheld when hyperinflammatory markers suggest a MAS-like phenotype (e.g., very high ferritin or rapidly rising IL-6/CRP).

Over the near term, we anticipate: analytical standardization of monocyte HLA-DR and whole-blood stimulation assays; translation of single-cell signatures into CLIA-ready panels; extension of spatial readouts to archived tissues using total-RNA–compatible chemistries; and composite risk models that outperform single biomarkers and enable allocation of immunoadjuvants to the subset most likely to benefit. These steps can convert heterogeneous, descriptive low-response states into tractable clinical entities with measurable entry criteria, reversible targets, and reproducible outcome gains.

## References

[1] Reyes M Filbin MR Bhattacharyya RP Sonny A Mehta A Billman K. Plasma from patients with bacterial sepsis or severe COVID-19 induces suppressive myeloid cell production from hematopoietic progenitors *in vitro* . Sci Trans Med. (2021) 13:eabe9599. doi: 10.1126/scitranslmed.abe9599, PMID: 34103408 PMC8432955

[2] Yao RQ Zhao PY Li ZX Liu YY Zheng LY Duan Y. Single-cell transcriptome profiling of sepsis identifies HLA-DR low S100A high monocytes with immunosuppressive function. Military Med Res. (2023) 10:27. doi: 10.1186/s40779-023-00462-y, PMID: 37337301 PMC10278311

[3] Leite GGF de Brabander J Michels EHA Butler JM Cremer OL Scicluna BP. Monocyte state 1 (MS1) cells in critically ill patients with sepsis or non-infectious conditions: association with disease course and host response. Crit Care. (2024) 28:88. doi: 10.1186/s13054-024-04868-5, PMID: 38504349 PMC10953179

[4] Wiersinga WJ van der Poll T Immunopathophysiology of human sepsis. Ebiomedicine. (2022) 86:104363–3. doi: 10.1016/j.ebiom.2022.104363, PMID: 36470832 PMC9783164

[5] de Roquetaillade C Dupuis C Faivre V Lukaszewicz AC Brumpt C Payen D. Monitoring of circulating monocyte HLA-DR expression in a large cohort of intensive care patients: relation with secondary infections. Ann Intensive Care. (2022) 12:39. doi: 10.1186/s13613-022-01010-y, PMID: 35526199 PMC9079217

[6] Haem Rahimi M Conti F Llitjos JF Fleurie A Cerro V Venet F. Monocyte HLA-DR expression as an enrollment biomarker in sepsis clinical trials: Evaluation of two sampling tubes and definition of respective clinical thresholds. Cytometry. Part B Clin cytometry. (2023) 104:468–70., PMID: 37226415 10.1002/cyto.b.22133

[7] Vacheron CH Lepape A Venet F Monneret G Gueyffier F Boutitie F. Granulocyte-macrophage colony-stimulating factor (GM-CSF) in patients presenting sepsis-induced immunosuppression: the GRID randomized controlled trial. J Crit Care. (2023) 78:154330. doi: 10.1016/j.jcrc.2023.154330, PMID: 37267804

[8] McKellar DW Mantri M Hinchman MM Parker JSL Sethupathy P Cosgrove BD. Spatial mapping of the total transcriptome by in *situ* polyadenylation. Nat Biotechnol. (2023) 41:513–20. doi: 10.1038/s41587-022-01517-6, PMID: 36329320 PMC10110464

[9] Mu P Li W Tran LP Li X SmT/SHM-seq: simultaneously capturing spatial transcriptome and microbiome information in plants. Trends Plant Sci. (2024). doi: 10.1016/j.tplants.2024.09.010, PMID: 39395879

[10] Lötstedt B Stražar M Xavier R Regev A Vickovic S Spatial host–microbiome sequencing reveals niches in the mouse gut. Nat Biotechnol. (2024) 42:1394–403., PMID: 37985876 10.1038/s41587-023-01988-1 PMC11392810

[11] Li Z Wang T Liu P Huang Y SpatialDM for rapid identification of spatially co-expressed ligand–receptor and revealing cell–cell communication patterns. Nat Commun. (2023) 14:3995. doi: 10.1038/s41467-023-39608-w, PMID: 37414760 PMC10325966

[12] Heitz M Ma Y Kubal S Schiebinger G Spatial transcriptomics brings new challenges and opportunities for trajectory inference. Annu Rev Biomed Data Sci. (2024) 8. doi: 10.1146/annurev-biodatasci-040324-030052, PMID: 39541229

[13] Clifton K Anant M Aihara G Atta L Aimiuwu OK Kebschull JM. STalign: Alignment of spatial transcriptomics data using diffeomorphic metric mapping. Nat Commun. (2023) 14:8123. doi: 10.1038/s41467-023-43915-7, PMID: 38065970 PMC10709594

[14] Zhao Y Li Y He Y Wu J Liu Y Li X. Stereo-seq V2: Spatial mapping of total RNA on FFPE sections with high resolution. Cell. (2025). doi: 10.1016/j.cell.2025.08.008, PMID: 40882628

[15] Robles-Remacho A Sanchez-Martin RM Diaz-Mochon JJ. Spatial transcriptomics: emerging technologies in tissue gene expression profiling. Analytical Chem. (2023) 95:15450–60. doi: 10.1021/acs.analchem.3c02029, PMID: 37814884 PMC10603609

[16] Chen A Liao S Cheng M Ma K Wu L Lai Y. Spatiotemporal transcriptomic atlas of mouse organogenesis using DNA nanoball-patterned arrays. Cell. (2022) 185:1777–1792.e21. doi: 10.1016/j.cell.2022.04.003, PMID: 35512705

[17] Liu R Liu J Cao Q Chu Y Chi H Zhang J. Identification of crucial genes through WGCNA in the progression of gastric cancer. J Cancer. (2024) 15:3284. doi: 10.7150/jca.95757, PMID: 38817876 PMC11134444

[18] Stuart T Srivastava A Madad S Lareau CA Satija R Single-cell chromatin state analysis with Signac. Nat Methods. (2021) 18:1333–41. doi: 10.1038/s41592-021-01282-5, PMID: 34725479 PMC9255697

[19] Tao Y Sun X Wang F. BiGATAE: a bipartite graph attention auto-encoder enhancing spatial domain identification from single-slice to multi-slices. Briefings Bioinf. (2024) 25:bbae045. doi: 10.1093/bib/bbae045, PMID: 38385877 PMC10883416

[20] Liu X Qu C Liu C Zhu N Huang H Teng F. StereoSiTE: a framework to spatially and quantitatively profile the cellular neighborhood organized iTME. GigaScience. (2024) 13:giae078. doi: 10.1093/gigascience/giae078, PMID: 39452614 PMC11503478

[21] Saarenpää S Shalev O Ashkenazy H Carlos V Lundberg DS Weigel D. Spatial metatranscriptomics resolves host–bacteria–fungi interactomes. Nat Biotechnol. (2024) 42:1384–93. 10.1038/s41587-023-01979-2 PMC11392817 37985875

[22] Wong-Rolle A Dong Q Zhu Y Divakar P Hor JL Kedei N. Spatial meta-transcriptomics reveal associations of intratumor bacteria burden with lung cancer cells showing a distinct oncogenic signature. J Immunotherapy Cancer. (2022) 10:e004698. doi: 10.1136/jitc-2022-004698, PMID: 35793869 PMC9260850

[23] Li X Xu H Du Z Cao Q Liu X Advances in the study of tertiary lymphoid structures in the immunotherapy of breast cancer. Front Oncol. (2024) 14:1382701. doi: 10.3389/fonc.2024.1382701, PMID: 38628669 PMC11018917

[24] Slim MA van Mourik N Bakkerus L Fuller K Acharya L Giannidis T. Towards personalized medicine: a scoping review of immunotherapy in sepsis. Crit Care. (2024) 28:183. doi: 10.1186/s13054-024-04964-6, PMID: 38807151 PMC11134696

[25] Joshi I Carney WP Rock EP. Utility of monocyte HLA-DR and rationale for therapeutic GM-CSF in sepsis immunoparalysis. Front Immunol. (2023) 14:1130214. doi: 10.3389/fimmu.2023.1130214, PMID: 36825018 PMC9942705

[26] Schott M León-Periñán D Splendiani E Strenger L Licha JR Pentimalli TM. Open-ST: High-resolution spatial transcriptomics in 3D. Cell. (2024) 187:3953–3972.e26. doi: 10.1016/j.cell.2024.05.055, PMID: 38917789

[27] Peng G Chi H Gao X Zhang J Song G Xie X. Identification and validation of neurotrophic factor-related genes signature in HNSCC to predict survival and immune landscapes. Front Genet. (2022) 13:1010044. doi: 10.3389/fgene.2022.1010044, PMID: 36406133 PMC9672384

[28] Qiu X Zhu DY Lu Y Yao J Jing Z Min KH. Spatiotemporal modeling of molecular holograms. Cell. (2024) 187:7351–7373.e61. doi: 10.1016/j.cell.2024.10.011, PMID: 39532097

[29] Benjamin K Bhandari A Kepple JD Qi R Shang Z Xing Y. Multiscale topology classifies cells in subcellular spatial transcriptomics. Nature. (2024) 630:943–9. doi: 10.1038/s41586-024-07563-1, PMID: 38898271 PMC11208150

[30] Cesaro G Nagai JS Gnoato N Chiodi A Tussardi G Klöker V. Advances and challenges in cell–cell communication inference: a comprehensive review of tools, resources, and future directions. Briefings Bioinf. (2025) 26:bbaf280. doi: 10.1093/bib/bbaf280, PMID: 40536815 PMC12204611

[31] Troulé K Petryszak R Cakir B Cranley J Harasty A Prete M. CellPhoneDB v5: inferring cell–cell communication from single-cell multiomics data. Nat Protoc. (2025), 1–29., PMID: 40133495 10.1038/s41596-024-01137-1

[32] Wilk AJ Shalek AK Holmes S Blish CA Comparative analysis of cell–cell communication at single-cell resolution. Nat Biotechnol. (2024) 42:470–83. doi: 10.1038/s41587-023-01782-z, PMID: 37169965 PMC10638471

[33] Dimitrov D Türei D Garrido-Rodriguez M Burmedi PL Nagai JS Boys C. Comparison of methods and resources for cell-cell communication inference from single-cell RNA-Seq data. Nat Commun. (2022) 13:3224. doi: 10.1038/s41467-022-30755-0, PMID: 35680885 PMC9184522

[34] Chen Y You Y Wei M Yang P Zhang Q Li X. Exploration of physical activity, sedentary behavior and insulin level among short sleepers. Front Endocrinol. (2024) 15:1371682. doi: 10.3389/fendo.2024.1371682, PMID: 39469577 PMC11513348

[35] Li Y Tan R Chen Y Liu Z Chen E Pan T. SC2sepsis: sepsis single-cell whole gene expression database. Database. (2022) 2022:baac061. doi: 10.1093/database/baac061, PMID: 35980286 PMC9387141

[36] Reyes M Filbin MR Bhattacharyya RP Billman K Eisenhaure T Hung DT. An immune-cell signature of bacterial sepsis. Nat Med. (2020) 26:333–40. doi: 10.1038/s41591-020-0752-4, PMID: 32066974 PMC7235950

[37] Yin J Chen Y Huang JL Yan L Kuang ZS Xue MM. Prognosis-related classification and dynamic monitoring of immune status in patients with sepsis: A prospective observational study. World J Emergency Med. (2021) 12:185. doi: 10.5847/wjem.j.1920-8642.2021.03.004, PMID: 34141032 PMC8188286

[38] Yende S Kellum JA Talisa VB Peck Palmer OM Chang CH Filbin MR. Long-term host immune response trajectories among hospitalized patients with sepsis. JAMA network Open. (2019) 2:e198686–e198686. doi: 10.1001/jamanetworkopen.2019.8686, PMID: 31390038 PMC6686981

[39] Mangioni D Peri AM Rossolini GM Viaggi B Perno CF Gori A. Toward rapid sepsis diagnosis and patient stratification: What’s new from microbiology and omics science. J Infect Dis. (2020) 221:1039–47., PMID: 31693109 10.1093/infdis/jiz585

[40] Komorowski M Green A Tatham KC Seymour C Antcliffe D Sepsis biomarkers and diagnostic tools with a focus on machine learning. EBioMedicine. (2022) 86. doi: 10.1016/j.ebiom.2022.104394, PMID: 36470834 PMC9783125

[41] Daix T Mathonnet A Brakenridge S Dequin PF Mira JP Berbille F. Intravenously administered interleukin-7 to reverse lymphopenia in patients with septic shock: a double-blind, randomized, placebo-controlled trial. Ann Intensive Care. (2023) 13:17. doi: 10.1186/s13613-023-01109-w, PMID: 36906875 PMC10008152

[42] Roquilly A Francois B Huet O Launey Y Lasocki S Weiss E. Interferon gamma-1b for the prevention of hospital-acquired pneumonia in critically ill patients: a phase 2, placebo-controlled randomized clinical trial. Intensive Care Med. (2023) 49:530–44. doi: 10.1007/s00134-023-07065-0, PMID: 37072597 PMC10112824

[43] Bouras M Tessier P Poulain C Schirr-Bonnans S Roquilly A Three-month outcomes and cost-effectiveness of interferon gamma-1b in critically ill patients: a secondary analysis of the PREV-HAP trial. J Intensive Care. (2024) 12:40. doi: 10.1186/s40560-024-00753-z, PMID: 39394183 PMC11468134

[44] Fung JST Wright RC Bharaj DK Alghamdi A Hesson D Delisle JS. Virus-specific T-cell therapy for prophylaxis and treatment of cytomegalovirus infections after transplantation: a scoping review. Clin Infect Dis. (2025), ciaf232. doi: 10.1093/cid/ciaf232, PMID: 40327446 PMC12596374

[45] Qiang C Qi Z Yi Q. Mechanisms of p2x7 receptor involvement in pain regulation: a literature review. Acta Med Mediterr. (2022) 38:1187–94.

[46] You Y Fu Y Li L Zhang Z Jia S Lu S. Systematic comparison of sequencing-based spatial transcriptomic methods. Nat Methods. (2024) 21:1743–54. doi: 10.1038/s41592-024-02325-3, PMID: 38965443 PMC11399101

[47] Fulci V. Fast analysis of Spatial Transcriptomics (FaST): an ultra lightweight and fast pipeline for the analysis of high resolution spatial transcriptomics. NAR Genomics Bioinf. (2025) 7:lqaf044. doi: 10.1093/nargab/lqaf044, PMID: 40248491 PMC12004221

[48] Zhang P Zhang H Tang J Ren Q Zhang J Chi H. The integrated single-cell analysis developed an immunogenic cell death signature to predict lung adenocarcinoma prognosis and immunotherapy. Aging (Albany NY). (2023) 15:10305. doi: 10.18632/aging.205077, PMID: 37796202 PMC10599752

[49] Ma H Srivastava S Ho SWT Xu C Lian BSX Ong X. Spatially resolved tumor ecosystems and cell states in gastric adenocarcinoma progression and evolution. Cancer Discov. (2025) 15:767–92. doi: 10.1158/2159-8290.CD-24-0605, PMID: 39774838 PMC11962405

[50] Liu H Liang Q Han L Single-cell multi-omics-based immune temporal network resolution in sepsis: unravelling molecular mechanisms and precise therapeutic targets. Front Immunol. (2025) 16:1616794. doi: 10.3389/fimmu.2025.1616794, PMID: 40861459 PMC12375439

[51] Browaeys R Saelens W Saeys Y. NicheNet: modeling intercellular communication by linking ligands to target genes. Nat Methods. (2020) 17:159–62. doi: 10.1038/s41592-019-0667-5, PMID: 31819264

[52] Jin S Guerrero-Juarez CF Zhang L Chang I Ramos R Kuan CH. Inference and analysis of cell-cell communication using CellChat. Nat Commun. (2021) 12:1088. doi: 10.1038/s41467-021-21246-9, PMID: 33597522 PMC7889871

[53] Palla G Spitzer H Klein M Fischer D Schaar AC Kuemmerle LB. Squidpy: a scalable framework for spatial omics analysis. Nat Methods. (2022) 19:171–8. doi: 10.1038/s41592-021-01358-2, PMID: 35102346 PMC8828470

[54] He S Su L Hu H Liu H Xiong J Gong X. Immunoregulatory functions and therapeutic potential of natural killer cell-derived extracellular vesicles in chronic diseases. Front Immunol. (2024) 14:1328094. doi: 10.3389/fimmu.2023.1328094, PMID: 38239346 PMC10795180

[55] Biancalani T Scalia G Buffoni L Avasthi R Lu Z Sanger A. Deep learning and alignment of spatially resolved single-cell transcriptomes with Tangram. Nat Methods. (2021) 18:1352–62. doi: 10.1038/s41592-021-01264-7, PMID: 34711971 PMC8566243

[56] Sun D Liu Z Li T Wu Q Wang C STRIDE: accurately decomposing and integrating spatial transcriptomics using single-cell RNA sequencing. Nucleic Acids Res. (2022) 50:e42–2. doi: 10.1093/nar/gkac150, PMID: 35253896 PMC9023289

[57] Cao Q Zhang Q Zhou KX Li YX Yu Y He ZX. Lung cancer screening study from a smoking population in Kunming. Eur Rev Med Pharmacol Sci. (2022) 26:7091–8. 10.26355/eurrev_202210_29894 36263557

[58] Duan B Chen S Cheng X Liu Q Multi-slice spatial transcriptome domain analysis with SpaDo. Genome Biol. (2024) 25:73. doi: 10.1186/s13059-024-03213-x, PMID: 38504325 PMC10949687

[59] Fu YC Das A Wang D Braun R Yi R scHolography: a computational method for single-cell spatial neighborhood reconstruction and analysis. Genome Biol. (2024) 25:164. doi: 10.1186/s13059-024-03299-3, PMID: 38915088 PMC11197379

[60] Baker EAG Schapiro D Dumitrascu B Vickovic S Regev A In silico tissue generation and power analysis for spatial omics. Nat Methods. (2023) 20:424–31. doi: 10.1038/s41592-023-01766-6, PMID: 36864197 PMC9998272

[61] Yan L Sun X. Benchmarking and integration of methods for deconvoluting spatial transcriptomic data. Bioinformatics. (2023) 39:btac805. doi: 10.1093/bioinformatics/btac805, PMID: 36515467 PMC9825747

[62] Gaspard-Boulinc LC Gortana L Walter T Barillot E Cavalli FMG Cell-type deconvolution methods for spatial transcriptomics. Nat Rev Genet. (2025), 1–19. doi: 10.1038/s41576-025-00845-y, PMID: 40369312

[63] Sang-Aram C Browaeys R Seurinck R Saeys Y Spotless, a reproducible pipeline for benchmarking cell type deconvolution in spatial transcriptomics. elife. (2024) 12:RP88431., PMID: 38787371 10.7554/eLife.88431 PMC11126312

[64] Dries R Zhu Q Dong R Eng CL Li H Liu K. Giotto: a toolbox for integrative analysis and visualization of spatial expression data. Genome Biol. (2021) 22:78. doi: 10.1186/s13059-021-02286-2, PMID: 33685491 PMC7938609

[65] Zahedi R Ghamsari R Argha A Macphillamy C Beheshti A Alizadehsani R. Deep learning in spatially resolved transcriptomics: a comprehensive technical view. Briefings Bioinf. (2024) 25:bbae082. doi: 10.1093/bib/bbae082, PMID: 38483255 PMC10939360

[66] Yan Z Fan KQ Zhang Q Wu X Chen Y Wu X. Comparative analysis of the performance of the large language models DeepSeek-V3, DeepSeek-R1, open AI-O3 mini and open AI-O3 mini high in urology. World J Urol. (2025) 43:416. doi: 10.1007/s00345-025-05757-4, PMID: 40622427 PMC12234633

[67] Armingol E Baghdassarian HM Lewis NE. The diversification of methods for studying cell–cell interactions and communication. Nat Rev Genet. (2024) 25:381–400. doi: 10.1038/s41576-023-00685-8, PMID: 38238518 PMC11139546

[68] Hussain A Moxley-Wyles B Bryan M Gordon-Weeks A Al-Obaidi I Sandhu C. Cancer vaccine trial evaluations: immunobridging and potential immunological endpoints. Immunotherapy Adv. (2025) 5:ltaf016., PMID: 40438385 10.1093/immadv/ltaf016 PMC12116883

[69] Mekata K Kyo M Tan M Shime N Hirohashi N Molecular endotypes in sepsis: integration of multicohort transcriptomics based on RNA sequencing. J Intensive Care. (2025) 13:30. doi: 10.1186/s40560-025-00802-1, PMID: 40448231 PMC12123803

[70] Atreya MR Huang M Moore AR Zheng H Hasin-Brumshtein Y Fitzgerald JC. Identification and transcriptomic assessment of latent profile pediatric septic shock phenotypes. Crit Care. (2024) 28:246. doi: 10.1186/s13054-024-05020-z, PMID: 39014377 PMC11253460

[71] Sounart H Lázár E Masarapu Y Wu J Várkonyi T Glasz T. Dual spatially resolved transcriptomics for human host–pathogen colocalization studies in FFPE tissue sections. Genome Biol. (2023) 24:237. doi: 10.1186/s13059-023-03080-y, PMID: 37858234 PMC10588020

[72] You Y Chen Y Zhang Q Hu X Li X Yang P. Systematic and meta-based evaluation of the relationship between the built environment and physical activity behaviors among older adults. PeerJ. (2023) 11:e16173. doi: 10.7717/peerj.16173, PMID: 37780389 PMC10538293

[73] Peronnet E Blein S Venet F Cerrato E Fleurie A Llitjos JF. Immune Profiling Panel gene set identifies critically ill patients with low monocyte human leukocyte antigen-Dr expression: preliminary results from the REAnimation low immune status marker (REALISM) study. Crit Care Med. (2023) 51:808–16. doi: 10.1097/CCM.0000000000005832, PMID: 36917594 PMC10187625

[74] Chenoweth JG Brandsma J Striegel DA Genzor P Chiyka E Blair PW. Sepsis endotypes identified by host gene expression across global cohorts. Commun Med. (2024) 4:120. doi: 10.1038/s43856-024-00542-7, PMID: 38890515 PMC11189468

[75] Das A Ariyakumar G Gupta N Kamdar S Barugahare A Deveson-Lucas D. Identifying immune signatures of sepsis to increase diagnostic accuracy in very preterm babies. Nat Commun. (2024) 15:388. doi: 10.1038/s41467-023-44387-5, PMID: 38195661 PMC10776581

[76] Cajander S Kox M Scicluna BP Weigand MA Mora RA Flohé SB. Profiling the dysregulated immune response in sepsis: overcoming challenges to achieve the goal of precision medicine. Lancet Respir Med. (2024) 12:305–22. doi: 10.1016/S2213-2600(23)00330-2, PMID: 38142698

[77] Di Marco F Nicola F Giannese F Saliu F Tonon G de Pretis S. Dual spatial host-bacterial gene expression in Mycobacterium abscessus respiratory infections. Commun Biol. (2024) 7:1287. doi: 10.1038/s42003-024-06929-5, PMID: 39384974 PMC11479615

[78] Monneret G Voirin N Richard JC Cour M Rimmelé T Garnier L. Monitoring monocyte HLA-DR expression and CD4+ T lymphocyte count in dexamethasone-treated severe COVID-19 patients. Ann Intensive Care. (2024) 14:76. doi: 10.1186/s13613-024-01310-5, PMID: 38762684 PMC11102415

[79] Silva EE Skon-Hegg C Badovinac VP Griffith TS The calm after the storm: implications of sepsis immunoparalysis on host immunity. J Immunol. (2023) 211:711–9. doi: 10.4049/jimmunol.2300171, PMID: 37603859 PMC10449360

[80] Liu W Xia L Peng Y Cao Q Xu K Luo H. Unraveling the significance of cuproptosis in hepatocellular carcinoma heterogeneity and tumor microenvironment through integrated single-cell sequencing and machine learning approaches. Discover Oncol. (2025) 16:900. doi: 10.1007/s12672-025-02696-9, PMID: 40411678 PMC12103433

[81] Leventogiannis K Kyriazopoulou E Antonakos N Kotsaki A Tsangaris I Markopoulou D. Toward personalized immunotherapy in sepsis: The PROVIDE randomized clinical trial. Cell Rep Med. (2022) 3. doi: 10.1016/j.xcrm.2022.100817, PMID: 36384100 PMC9729870

[82] Bodinier M Peronnet E Llitjos JF Kreitmann L Brengel-Pesce K Rimmelé T. Integrated clustering of multiple immune marker trajectories reveals different immunotypes in severely injured patients. Crit Care. (2024) 28:240. doi: 10.1186/s13054-024-04990-4, PMID: 39010113 PMC11247757

[83] Bode C Weis S Sauer A Wendel-Garcia P David S Targeting the host response in sepsis: current approaches and future evidence. Crit Care. (2023) 27:478. doi: 10.1186/s13054-023-04762-6, PMID: 38057824 PMC10698949

[84] Oelen R de Vries DH Brugge H Gordon MG Vochteloo M Single-cell RNA-sequencing of peripheral blood mononuclear cells reveals widespread, context-specific gene expression regulation upon pathogenic exposure. Nat Commun. (2022) 13:3267. doi: 10.1038/s41467-022-30893-5, PMID: 35672358 PMC9174272

[85] Shi W Zhang J Huang S Fan Q Cao J Zeng J. Next-generation sequencing-based spatial transcriptomics: A perspective from barcoding chemistry. JACS Au. (2024) 4:1723–43. doi: 10.1021/jacsau.4c00118, PMID: 38818076 PMC11134576

[86] Jang S Lee EJ Park S Lim H Ahn B Huh Y. Spatial host-microbiome profiling demonstrates bacterial-associated host transcriptional alterations in pediatric ileal Crohn’s disease. Microbiome. (2025) 13:189. doi: 10.1186/s40168-025-02178-8, PMID: 40849632 PMC12374449

[87] Kajihara K Yan D Seim GL Little-Hooy H Kang J Chen C. Systemic cytokines drive conserved severity-associated myeloid responses across bacterial and viral infections. Commun Biol. (2025) 8:1096. doi: 10.1038/s42003-025-08407-y, PMID: 40702253 PMC12287453

[88] Wang Z Zhang W Chen L Lu X Tu Y Lymphopenia in sepsis: a narrative review. Crit Care. (2024) 28:315. doi: 10.1186/s13054-024-05099-4, PMID: 39304908 PMC11414153

